# Urbanicity—Perspectives from Neuroscience and Public Health: A Scoping Review

**DOI:** 10.3390/ijerph20010688

**Published:** 2022-12-30

**Authors:** Ben Senkler, Julius Freymueller, Susanne Lopez Lumbi, Claudia Hornberg, Hannah-Lea Schmid, Kristina Hennig-Fast, Gernot Horstmann, Timothy Mc Call

**Affiliations:** 1 Sustainable Environmental Health Sciences, Medical School OWL, Bielefeld University, 33615 Bielefeld, Germany; 2 Psychotherapy and Psychosomatics, Department Psychiatry, Medical School OWL, Bielefeld University, 33615 Bielefeld, Germany; 3 Neurocognitive Psychology, Department Psychology, Faculty of Psychology and Sport Science, Bielefeld University, 33615 Bielefeld, Germany

**Keywords:** Urban Health, urban brain, urban planning, Mental Health, brain structure, Public Health, Neuroscience

## Abstract

Urban residency is associated with exposure to environmental factors, which can influence health in many ways. Neuroscientific research, as well as Public Health research, aim towards broadening evidence in the field of Urban Health. However, it is unclear whether the association between urban living and mental illnesses is causal rather than explainable by other selective effects. This review seeks to gather information on the current evidence regarding urban living and neurological outcomes to demonstrate how Public Health and Neuroscience could complement each other in the field of Urban Health. A scoping review was conducted in four electronic databases according to the PRISMA-statement guidelines. 25 empirical studies were included. Outcomes such as schizophrenia and psychotic disorders, social and cognitive functioning were scrutinised. Evidence was found for alteration of brain functioning and brain structure. Most studies researching cognitive functioning or cognitive decline displayed possible protective effects of urban living compared to rural living. The different study designs in Public Health and Neuroscience could profit from each other. Although the comparability of studies is limited by the inconsistent assessments of urbanity. Synergies and potentials to combine aspects of Public Health and Neuroscience in the field of Urban Health to improve population health became apparent.

## 1. Introduction

Inarguably, the variability of environment in which populations live is one of the major determinants of human health. Each year, the environmental burden of disease is estimated to be about 22% of the total burden, underscoring the importance of the scientific debate [1]. Barton and Grant’s human ecology model [2] discusses the many ways in which the environment can influence the health of different populations. Their research refers to many complex determinants of health, such as lifestyle, community, and local economy, or the different activities of people. At the same time, they also show how the built environment, the natural environment, and the global ecosystem determine health within settlements [2]. 

Fundamental changes in the human living environments are evident. Urban regions are growing while rural populations are shrinking [3]. The United Nations predict that 68% of the world’s population will live in cities by 2050. The World Health Organization (WHO) describes this change as the largest migration in history and points to the global importance of equity and health in urban environments [4]. As such, cities require a special focus within the field of environmental health. On one hand, cities are regarded as environments that offer a variety of health benefits, such as diverse opportunities for education, a variety of workplaces, and leisure activities [5]. On the other hand, however, urban environments are linked to specific risk factors for health. Urban dwellers experience an increased exposure to air pollutants [6], (road traffic) noise [7], or urban heat islands [8]. Some of these risk factors are associated with cardiovascular [9,10] or respiratory diseases [11]. In recent years, an increasing number of people were exposed to urban environmental noise, which is associated with an increased burden of mental illnesses [12]. For example, studies have identified a link between sleep disturbances caused by road noise and the risk of developing depression [13,14]. Furthermore, when rural and urban residents are compared, it becomes evident that individuals in urban populations are more frequently affected by mental illnesses, such as schizophrenia or other non-affective psychosis, depression or anxiety [15,16]

The interdisciplinary Public Health subfield, “Urban Health” addresses these specific environmental issues. Urban Health assesses the impact of living in urban areas (urbanicity), [17]) and in particular, its association with health. It applies theoretical and practical approaches in order to systematically measure the health of urban populations via the assessment of urban epidemiological determinants, health states, and deprivation [18]. In addition, Urban Health addresses planning and disease prevention, health protection, and urban governance processes. The different sciences regarding health are dependent on a high degree of cooperation to make contributions to Public Health. As such, methods and contents of psychology or epidemiology, along with many other disciplines, can be integrated [19]. In this way, Public Health attempts to connect findings from disciplines that previously conducted research separately from each other and to ask questions that cross these fields [20]. The strong population-level focus of Public Health results in specific approaches. Thus, Public Health research in the context of urban environment and mental health frequently uses social epidemiological and therefore empirical methods [21]. In many aspects, without experimental study designs it is difficult to establish mechanistic pathways [22]. Therefore, with respect to the increased number of cases of mental illnesses in cities, one cannot sufficiently explain whether the effects are causal, or subject to a selection effect [23]. This means that mentally unstable populations may relocate more frequently to cities to take advantage of better health care or to escape possible stigmatization in the rural milieu. 

To better understand the neural mechanisms behind urbanicity and mental health, the field of Neuroscience has increasingly focused on neuronal systems [24,25]. In this regard, some scientists assume that the influence of cities could be exerted through stress reactions [23]. Different forms of social stress, such as isolation or density stress, could be influential variables for health-related brain alterations or cognitive changes. However, other possible causes for the increased incidence of mental illness in cities are also being discussed, such as the neurotoxicity of certain air pollutants [26,27]. For example, cognitive impairment has been linked to longterm ozone exposure [28]. The brain represents an important target of investigation in the consideration of environmental changes, because it has the ability to adapt to the given influences through neuronal plasticity, which in turn can be measured by imaging methods [29]. To combine the results of the individual sciences regarding urban mental health, the research field of “neurourbanism” has emerged [23], in which the various aspects of living in urban environments are studied and applied in research and practice. Although neurourbanism represents an amalgamation of many disciplines in which urban planners, sociologists, physicians, and other professions contribute to the topic, Public Health is not specifically mentioned as a discipline. Although Neuroscience and Public Health as disciplines differ widely in foci of interest and approaches, complementarities also appear to link the two disciplines.

The review aims to identify the relationship between urban life and changes in neuronal systems and, based on the results, to identify the ways in which Neuroscience and Public Health differ and complement each other to further the field of neurourbanism.

## 2. Materials and Methods

A scoping review was conducted according to the Preferred Reporting Items for Systematic Reviews and Meta-Analyses (PRISMA) statement guidelines [30]. The search strategy was designed according to the PICO framework. The searches were conducted in the electronic databases Medline (Pubmed), PsycInfo, Embase, and CINAHL, and were last updated on the 13th of January 2022. The review was not registered, and no protocol was prepared. 

The respective search strategies for each database can be seen in the Supplemental Material (Table S1). The criteria used for inclusion and exclusion of studies in the screening process can be seen in Table 1. To be included, studies had to measure current urbanicity or urban upbringing of the study population. Studies measuring at least one primary neuronal indicator (e.g., connectivity or activity of neuronal systems) or a secondary neuronal indicator, e.g., cognition, were included. Studies assessing outcomes such as stress via self-report and without objective parameters were excluded. Primary studies assessing these measurements were included, while reviews or theory papers were excluded. In order to look at urbanity in a holistic way first, without looking at individual partial aspects, studies researching other environmental exposures such as air pollution or environmental noise were excluded. Urban childhood was considered by urban upbringing in the first 15 years. Due to the consideration of longterm effects, studies with participants of around 15 years and older were considered (cf. [24]). To reflect the timeliness of the rapidly evolving field of neurourbanism, only studies between 2016 and 2021 were included. The search was restricted to studies which were available in English or German.

The review process included several stages. After removing duplicates, two independent authors (B.S. & J.F.) first screened the titles and abstracts of the publications to determine their relevance. Second, a full text screening was conducted for studies matching the inclusion criteria (B.S. & J.F.). Dissent regarding the inclusion or exclusion of studies was resolved by a third author (T.M.C. or S.L.L.). The data collection process was conducted by two independent authors (B.S. & J.F.). All main outcomes of the studies were collected and further the study design, methodology, limitations (author identified) and limitations (self- identified) were recorded (Table S2).

## 3. Results

**Figure 1:**
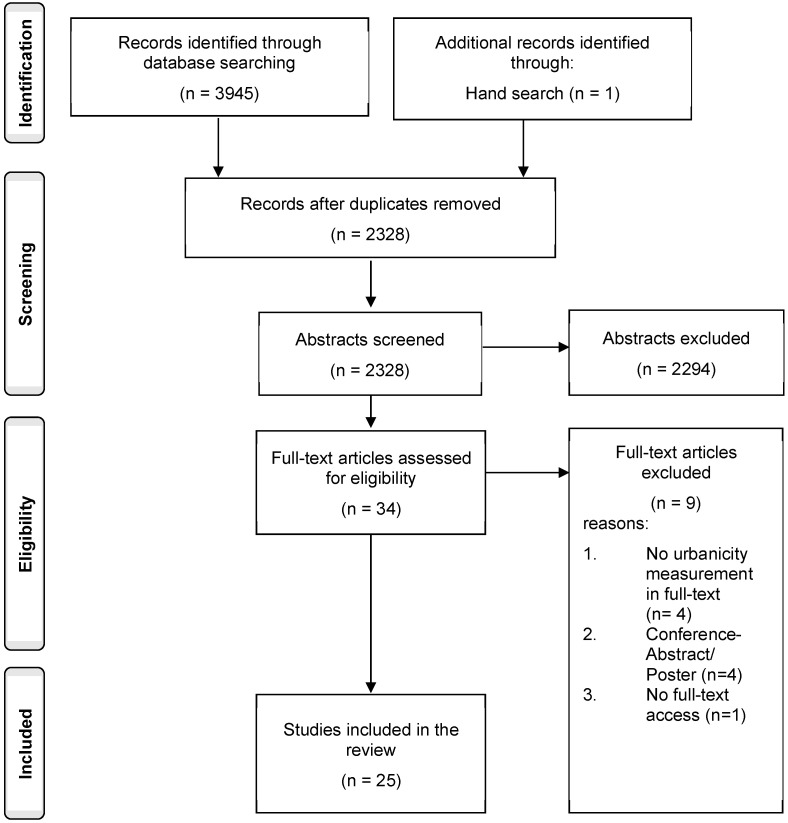
Flowchart of study selection progress.

In total, 3945 publications were retrieved via database search of which 1618 duplicates were removed. One study was found via hand search. A total of 2328 abstracts and titles were screened, of which 2294 were excluded. This resulted in the full text screening of 34 studies. Of these, 25 studies were included in the review. For further details regarding the screening process see Figure 1, below. Table S2 presents the characteristics of the included publications.

### 3.1. Studies Included

The sample sizes of the 25 included studies varied between 31 and 19,667 participants, summing up to 119,858 subjects across the studies. All studies included male and female participants. 

Differences in the study design of the individual studies were identified. A cross-sectional study design was used in 13 studies. [31,32,33,34,35,36,37,38,39,40,41,42,43]. Another six studies examined the population in a longitudinal design [44,45,46,47,48,49]. Six studies employed an experimental design [50,51,52,53,54,55]. Two studies used the same study population which also included a control group [34,35].

Furthermore, 13 of the included studies focused on adults aged 50 and above [32,33,38,39,41,42,43,44,45,46,48,49,55]. Other papers studied adults under 50 years [31,34,35,37,51,53,54]. One study included adults between 18 and 55 years [52] and two studies analyzed adolescents around the age of 15 [36,50]. Further details regarding the age of the study populations are presented in Table S2.

Moreover, five studies were conducted in China [41,44,46,47,49], four in Germany [31,37,53,54], three in Ireland [32,33,55] and five in the Netherlands [34,35,45,50,51]. One study each came from the Czech Republic [39], UK [40], India [42], Canada [48], and Mexico [38]. Some publications included participants from multiple countries. One study was conducted in China and India [43], one in UK, France, Germany, 

### 3.2. Methods of Included Studies 

Methodological approaches in the studies were heterogeneous in accordance with the different disciplines included. Nine neuroscientific studies used functional magnetic resonance imaging (fMRI) to investigate neuronal changes [31,34,35,36,37,51,52,53,54]. One of the neuroscientific studies employed the specific stress inducing paradigm “Montreal imaging stress task” [53], while Evans, Huizink et al. [50] used their own stress tasks consisting of mental arithmetic tasks, public speaking tasks, and a computer mathematics task in front of the test leader, while measuring electrocardio-activity. Other fMRI studies included the trust game or the desire-reason-dilemma paradigm [51,54]. Population based studies with Public Health focus mostly used cognitive functioning tests in order to determine cognitive differences [32,33,36,38,39,40,41,42,43,44,45,46,47,48,49,55]. The Mini Mental State Examination (MMSE) in different versions was most frequently used to assess basic cognitive parameters [32,33,39,40,44,45,46,47,48,49]. In addition to the MMSE, Cassarino et al. [32,33] used the Montreal Cognitive State examination (MoCA), immediate and delayed recall, the verbal fluency test, and the Color Trail Making Test (CTT). Furthermore, Saenz et al. [38] applied the “Cross Cultural Cognitive Examination”, while Kühn et al. [36] used items from the Wechsler Intelligence Test and elements of the Cambridge Neuropsychological Test Automated Battery to assess cognitive functioning. Another study measured hormonal and physiological biomarkers to determine neuronal activity (hair or salivary cortisol, heart rate) [50].

### 3.3. Assessment of Urbanicity

Urbanicity was defined and assessed heterogeneously throughout the studies. Most of the studies focused on population size or density as an urbanicity indicator. Additionally, studies differentiated between current urbanicity of their participants, urbanicity by childhood residence or change of urbanicity through the life course.

The measurement of urbanicity by population size differed slightly. Two Irish studies [33,55] differentiated between three categories (i.e., urban: Dublin area (population > 1,000,000), other settlements: population 1500–200,000, and rural: population < 1500). Two studies [53,54] used three categories as well but employed other cut-off-values (i.e., small town or community (population < 20,000), middle town (population 20,000–100,000) and big city (population > 100,000)). Stepankova et al. [39] distinguished between the city of Prague, towns with 20,000–50,000 inhabitants and villages with <5000 inhabitants. Saenz et al. [38] distinguished between four categories to assess urbanicity of the participants residence ((a) >100,000 residents, (b) 15,000–99,999 residents, (c) 2500–14,999 residents, (d) <2500 residents). Another study categorized rural residence as living in a community with <2500 individuals [48]. 

Studies measuring urbanicity by population density also developed different categories. Cassarino et al. [32] used six categories based on persons per hectare ranging from 1 very low (<0.5 people per hectare) to 6 very high (>50 persons per hectare). Wu et al. [40] distinguished between three categories which were derived from the 2011 Rural/Urban Classification for Small Area Geographies. The first category included major conurbations (mean population density of 35.5 people per hectare) and minor conurbations (22.6 people per hectare), the second category referred to cities and towns (mean population density of 16.5 people per hectare), and the third category included towns and fringes (mean population density of 5.9 people per hectare) as well as villages and dispersed (mean population density of 0.5 people per hectare). Two studies [45,50] measured urbanicity on a five-category scale based on the surrounding address density. The categories ranged from 0, very rural defined as <500 addresses per km^2^ up to 4, very urban and >2500 addresses per km^2^.

One study reported urbanicity as a dichotomous variable based on self-reported residence (urban/rural) [44], while another study used the same dichotomous variable (urban/rural) but categorized their participants residential neighbourhood according to the Chinese National Bureau of Statistics’ definition of urban and rural [49]. Others reported residence as well as migration status. Xu et al. [47] used four categories with rural, urban, rural-to-urban and urban-to-rural comparing residence at birth with current residence based on self-report of the participants. Xie et al. [46] reported rural residence and rural-to-urban migration based on a Chinese household registration system (hukou status). Three studies [41,42,43] reported six categories (urban residents, rural residents, rural-to-urban migrants, urban-to-urban migrants, rural-to-rural migrants, urban-to-rural migrants) based on self-report. Xu, Dupre et al. [41] also reported the age at migration.

Seven studies developed scores regarding childhood urbanicity using population size or density. Two studies [31,37] assessed childhood urbanicity for the first 15 years of life. The authors used three categories (rural area (<10,000 inhabitants, value 1), small cities (10,000–100,000 inhabitants, value 2) and large cities/metropolitan centres (>100,000 inhabitants, value 3)) and aggregated each value for the first 15 years of life to a score between 15 and 45, where higher value represents higher level of urbanicity. Reed et al. [52] used the same categories but multiplied the maximal urbanicity score in a five-year period for the first 15 years of life. Two studies [34,35] used the five-category scale based on surrounding address density. Each category was assigned a value (1–5), ascending with higher density. The authors aggregated the average urbanicity for the periods between 0–4 years, 5–9 years and 10–14 years as childhood urbanicity. Lemmers-Jansen et al. [51] utilized the same scale for the first 15 years of life yet used a dichotomous variable reporting lower urbanicity (<2500 addresses per km^2^) and higher urbanicity (>2500 addresses per km^2^). One study matched adolescents who lived in a rural area for the first 15 years of their life with adolescents living in a town or city with a population over 100,000 for the first 15 years of their life [36]. 

### 3.4. Health Outcomes

The selected studies related neural indicators to various health outcomes. Studies with neuroscientific focus investigated neural mechanisms that relate to certain health outcomes, while studies with Public Health focus researched cognitive functioning. In general, the studies focused on schizophrenia, social functioning, depression/anxiety, or cognition.

#### 3.4.1. Schizophrenia/Psychotic Disorders

One identified category of investigation is the link between urbanicity and psychotic disorders. For example, Besteher et al. [31] found that cortical thickness in the areas of dorsolateral prefrontal cortex, bilateral medial prefrontal cortices, as well as left superior temporal cortex, left parahippocampal cortex, and bilateral medial parietal cortices/precuneus negatively correlates with urbanicity score. These brain regions overlap with brain regions that also contribute to the development of schizophrenia. Thus, Besteher et al. [31] described that the findings may indicate how structural changes in the brain provide a possible mechanism for the association between urbanicity and increased risk of psychotic disorders. However, Frissen et al. [34] also investigated in the association to psychotic disorders and found no link between urbanicity and cortical thickness, neither in healthy subjects nor for individuals at high genetic risk. Nevertheless, the authors emphasized that the absence of structural alterations does not automatically imply absence of functional alterations.

In addition to the consideration of cortical thickness, gray matter (GM) volume may be a possible variable in the association between psychotic disorders and exposure to urbanicity [35,36,37]. Frissen et al. [35] found a correlation between urban upbringing and generally lower GM volume in male participants with psychotic disorders. The authors state, that the reduction of GM is closely related to psychotic disorders. Another study found lower GM volume and changes in white matter structures in healthy individuals who grew up in a city compared to rural populations, specifically for the bilateral dorsolateral prefrontal cortex and the right inferior parietal lobe, the precuneus and superior longitudinal fasciculus [37]. Here, their findings pointed to a pathophysiological mechanism by which urban upbringing may affect brain maturation, leading to an increased risk for (first episode) schizophrenia. Likewise, Kühn et al. [36] measured lower GM volume in the left hippocampal formation of adolescents with an urban upbringing when compared with adolescents brought up in rural areas. The authors assumed that the hippocampus is subject to modulation by environmental factors [36]. At the same time, the lower hippocampal formation volume could be a risk factor for schizophrenia and the results obtained could thus provide evidence for a causal link [36].

In addition to the morphological associations with psychotic disorders, differences in brain activity were also found and associated with this group of disorders [52,53,54]. Reed et al. [52] found that participants with urban residence showed changes in three selected dopamine genes, as well as altered prefrontal activation in comparison to rural dwellers. The authors speculated that the observed interaction between dopamine genes and the urban environment may lead to altered cortical dopamine availability. The affected dopamine receptor D1 and decreased dopamine levels are found in patients with schizophrenia. In addition, Krämer et al. [54] found altered activation or modulation capability of the midbrain (ventral tegmental area (VTA)) dopamine system, as well as increased activation of the amygdala, orbitofrontal, and pregenual anterior cingulate cortex (pgACC) during the desire-reason paradigm in city dwellers. The authors linked the changes in the activation of the mesolimbic dopamine system to psychotic disorders and described that this specific dysregulation could be related to the pathogenesis and pathophysiology of schizophrenia [54].

#### 3.4.2. Social Functioning

Lemmers-Jansen et al. [51] found through their social experiment via a trust game that urban exposure during upbringing influences the trust in cooperation in a way, that psychotic patients from higher urbanity-scores fail to compensate their initial distrust. The authors pointed out that the amygdala, which in this case shows an altered activity, is crucial for performing several tasks that contribute to the healthy functioning of emotion and valence processing, reward learning memory and stress responsiveness. Dysfunction of this area can lead to a lack of social apprehension. Krämer et al. [54] showed that the detected activity of the ventral medial prefrontal cortex (vmPFC), and more precisely the pgACC, correlates with differences in urbanicity among subjects. Here, the authors highlighted that the vmPFC is involved in the integration of memory, social cognition, emotion, and reward. The pgACC is likewise involved in the regulation of emotional conflict and influences the inhibition of the hypothalamic-pituitary-adrenal (HPA) axis responses to psychogenic stressors [54].

#### 3.4.3. Depression/Anxiety

Richter et al. [53] found increased activity in the amygdala-hippocampus complex in participants with higher urbanicity scores. The authors pointed out that these effects are also frequently found in correlation with stress-related disorders such as depression. Similarly, Krämer et al. [54] presented a possible link between urbanicity and the development of depression. Here, the authors explained that the changes of the dopamine system, which they found in the midbrain (VTA), are related to the pathophysiology of depression. However, Evans, Huizink et al. [50] investigated the relationship between depressive or anxiety symptoms and the city via the function of the HPA axis and did not find any correlation indicating an altered likelihood of disease. 

#### 3.4.4. Cognitive Functioning and Cognitive Impairment 

All studies focusing on cognitive functioning and cognitive impairment analyzed older adults aged 60 or above, with the exception of Kühn et al. [36] who analyzed cognitive functioning in adolescents. Cognitive functioning is observed in different contexts of urbanicity (i.e., current or childhood urbanicity). Regarding current urbanicity, Cassarino et al. [33] found that urban residence was significantly associated with better global cognition compared to rural residence when assessed via the MMSE (b = −0.287, *p* < 0.001, ref. group: urban residence) and MoCA (beta coefficient (b) = −0.442, *p* < 0.01, ref. group urban residence). Furthermore, rural dwellers performed worse in areas such as verbal fluency (b = −1.829, *p* < 0.001, ref. group: urban residence), the picture memory test (Incidence Ratio Rate (IRR) = 0.987, *p* < 0.05, ref. group: urban residence), and had a higher completion time for further test procedures than urban residents. Further investigations in a later study with the same cohort showed similar associations when analyzing urban density and cognitive impairment [32]. Participants from areas with a higher population density performed better in all tests except for the CTT1 and CTT2 [32]. Participants from communities with <2500 inhabitants performed significantly worse than participants with a community size > 100,000 inhabitants in the categories verbal learning (b = −0.19, *p* < 0.001, ref. group: community size: > 100,000), verbal memory (b = −0.11, *p* < 0.001, ref. group: community size: > 100,000), verbal fluency (b = −0.17, *p* < 0.001, ref. group: community size > 100,000), orientation (b = −0.17, *p* < 0.001, ref. group: community size > 100,000), and attention (b = −0.27, *p* < 0.001, ref. group: community size > 100,000) [38]. In the study conducted by Stepankova et al. [39] Prague residents performed consistently better in all cognition tests than residents from other regions. In another study, participants from rural areas had significantly higher odds of cognitive impairment than participants from urban conurbations in the UK [40]. The authors also assessed the micro-scale environment and found that features of a poor-quality environment such as graffiti and broken windows seemed to play a role in the cognitive impairment of rural and urban residents. A poor-quality environment was associated with nearly twice higher odds of cognitive impairment in urban conurbations (Odds Ratio (OR) = 1.88; 95% Confidence Interval (CI): 1.18, 2.97). 

Another study analyzed cognitive decline via the modified MMSE over a time span of ten years in subsamples of people with dementia, without dementia, and with unknown dementia status [48]. Participants with an urban residence had significantly better initial cognitive functioning in the subsample of people with dementia and the subsample with unknown dementia status, but these differences were nullified after ten years. In the study conducted by Hou et al. [44] cognitive impairment in Chinese participants was studied based on the Chinese Version of the MMSE and categorized into no cognitive impairment, mild cognitive impairment and severe cognitive impairment. Urban residents were significantly less likely to enter mild cognitive impairment but were marginally more likely to change to severe cognitive impairment from mild impairment than people living in rural areas. Xiang et al. [49] found that in their sample of elder Chinese participants urban residents had a higher cognitive function at baseline (b = −0.822, *p* < 0.001) but also showed potentially faster decline (b = 0.320, *p* < 0.05) over the four years of assessment. Wörn et al. [45] reported a positive linear relationship between urbanicity and cognitive function when measured with the MMSE (b URB = 0.15, *p* = 0.001) and the 15-word test (b URB = 0.18, *p* = 0.004) over the course of six years. The rate of cognitive decline did not differ significantly between the researched populations. 

Two studies analyzed possible correlations between parts of cognitive performance and urbanicity. Kühn et al. [36] included the investigation of residential status while growing up and spatial processing via the Block Design test and the Spatial Working Memory task in their study. Adolescents with a rural upbringing performed significantly better in the Block Design test than their urban counterparts (mean score rural = 10.98, mean city = 9.56; F (1,83) = 8.06, *p* = 0.006), while there were no differences regarding the Spatial Working memory task [36]. Hirst et al. [55] examined multisensory perception and the correlation with current and childhood urbanicity in older adults. Multisensory perception was assessed with the sound-induced flash illusion measuring whether a visual flash combined with multiple audio cues is perceived as multiple visual flashes. Participants with a rural childhood residence were more susceptible to the illusion at longer sound-onset asynchronies than participants with an urban childhood residence [55].

Another context in which cognitive functioning was assessed was migration between rural and urban areas [41,42,43,46,47]. Xie et al. [46] examined cognitive performance and considered whether people changed their residence from rural to urban areas during the aging process. The authors found that women who migrated to urban areas achieved better scores for total cognition (b = 0.77, *p* < 0.001) and mental status (b = 0.68, *p* < 0.001) domains than non-migrant women. Such an association could not be observed for men. Xu, Vorderstrasse et al. [43] found that women with an urban residence had significantly better cognitive function than their counterparts living in rural areas (b = −1.96, *p* < 0.001 (China); b = −1.66, *p* < 0.001 (India), ref. group: urban residents) and the rural-to-rural-migrants (b = −2.60, *p* < 0.001 (China); b = −1.61, *p* < 0.001 (India), ref. group: urban residents) in the Chinese and Indian cohort. The researchers also reported that men living in an urban area had better cognitive function than rural male residents (b = −1.31, *p* < 0.001) within the Chinese subsample and rural-to-rural migrants (b = −1.54, *p* < 0.05) in the Indian subsample [43].

Xu et al. [47] examined cognitive decline over a time span of twelve years and found that regardless of the type of migration there was a deterioration in cognitive performance as people aged. However, rural-to-urban (b = 0.50, *p* < 0.01) and rural residents (b = 0.42, *p* < 0.01) had a higher level of cognition at baseline but showed a faster decline in cognitive function than urban residents. Xu, Dupre et al. [41] found that after adjusting for all covariates, rural-to-urban migrants had a significant better cognitive function than urban residents (b = 0.07, *p* < 0.05), but rural-to-rural migrants had a worse cognitive function than participants from urban areas (b = −0.11, *p* < 0.001). A similar study conducted in India found no significant differences in the fully adjusted model [42].

## 4. Discussion

The aim of this review was to examine the impact of urban living on neural systems in order to discuss how the disciplines of Neuroscience and Public Health in the field of Urban Health differ, but also complement each other, and could be synergistic in both, science and practice. 

The results demonstrate that urban living shows associations with brain structural differences and brain activity changes. Both are related to various health outcomes. At the same time, in many cases better cognitive performance is observed in urban areas. When current cognitive performance was assessed between urban residents and rural residents, urban dwellers frequently performed better than rural participants [32,33,38,39,41,43]. Most of the included longitudinal studies regarding cognitive functioning and cognitive impairment found, that living in or moving to an urban environment might have protective effects on cognitive decline for the elderly population in general or in some subpopulations [33,40,41,45,46,47]. Some studies had non-significant or inconsistent results [44,48]. There is also some evidence from an experimental study that sensory function seems to be influenced by urban living [55]. Regarding cognitive functioning in older adults, Cassarino & Setti [56] stated in their review that better sensory processing in urban older adults might be due to the higher exposure to multiple stimuli in cities. 

A possible neural mechanism explaining the effect of built environments on cognitive performance could be through functional connectivity of brain networks. Subjects in a study comparing neuronal connectivity displayed higher functional connectivity of certain neuronal networks during the presentation of photographs of natural environments in comparison to photographs of built environments [57]. Study participants showed lower functional connectivity during the natural environment condition compared to the built environment condition, depending on the years that individuals spent in major cities during their upbringing. Thus, the results of Kühn et al. [57] support the idea that neural mechanisms are influenced by living in the city or the countryside and therefore could be an important part in explaining the effects between cognition and city life. 

In contrast to the epidemiological research, the experimental studies showed that living and growing up in the city can possibly influence brain activity and brain structure of certain brain regions in adverse ways. Brain regions effected were for instance the cortical thickness in different areas such as the dorsolateral prefrontal cortex, bilateral medial prefrontal cortices, as well as left superior temporal cortex, left parahippocampal cortex or the reduction of GM volume in the left hippocampal formation [31,34,35,36]. Differences in brain activity were frequently found in the mesolimbic system or the amygdala [53,54] as well as effects of urbanicity on selected dopamine genes [52]. The detected changes in neuronal structures or activities were related to different health outcomes. Some of the included studies established a connection to the development of depression and anxiety disorders, or social functioning, whereas the largest part identified changes in brain regions that are also observed in the development of schizophrenia and non-affective psychosis.

Although the majority of the studies found evidence to support the impact of urban environments on neuronal systems, contradictory results can also be found. While Besteher et al. [31] found a significant association between urban upbringing with lower cortical thickness, the studies by Frissen et al. [34] did not confirm these results. Similarly, Evans, Huizink et al. [50], who examined the HPA axis by studying stress hormones in hair and saliva of adolescents did not find significant associations between depression and anxiety and living in the city. In this regard, the findings of Evans, Huizink et al. [50] are consistent with previous studies that also targeted stress hormones in hair and saliva of children [58,59]. Another study identified a significant change in HPA axis reactivity in adults from urban populations [60], thus contradicting the findings from Evans, Huizink et al. [50].

Nevertheless, it is important to emphasize that there is only some evidence of a causal effect, but this needs to be investigated further. Mental disorders are highly socially mediated and constructed. Consequently, they are a socio-medical phenomenon that cannot be diagnosed via a biomarker or on the basis of a brain image at the current state of research [61]. The neuroscientific studies, if they depict the actual effect, can only explain a part of a multicausal effect, due to the experimental study design.

However, the majority of identified results between 2016–2021 are largely consistent with prior research. Lederbogen et al. [25] found increased activity of the pgACC and amygdala in urban residents compared to rural residents among 32 healthy participants. In the measurement, the subjects were exposed to a stress inducing paradigm. Imaging the brain areas of 110 healthy subjects, Haddad et al. [24] found a strong inverse correlation between early life urbanicity and GM volume in the right dorsolateral prefrontal cortex. The researchers found a negative correlation of early life urbanicity and GM volumes in the pgACC in men only. These findings were linked to an increased risk of schizophrenia [24]. Thus, the previous results are consistent with the majority of the latest findings. Regarding the underlying causes, Krabbendam et al. [62] offer a variety of possible explanations for the correlation between urban living and altered health indicators, such as lack of natural space, social stress, or selective migration. Nevertheless, the authors point out, that it is necessary to interpret the results regarding neuronal functioning and brain structure cautiously, due to the fact that they only offer correlations.

The present scoping review was able to present some evidence for health benefits as well as health risks for living in the city. Since there is no clear evidence on what elements of urban life are specifically associated with changing health conditions, there is a need to explore and address the underlying pathways in research. Only then targeted prevention and health promotion in the urban environment can take place. 

At the same time, however, there are also methodological differences in the studies that influence the comparability of the results. First, there was a large variance in the assessment of urbanicity. Some studies used the parameter of population density or number of inhabitants as a basis and verified it by register data of residential addresses [32,38,39,40,45,50]. Conversely, some studies obtained participant-related data on urbanicity from interviews and questionnaires. These studies mostly referred to the number of inhabitants of the place of residence or the subjective opinion of the participants whether they considered their place of residence urban or rural [33,44,55]. Second, the studies employed different cut-off-values for urbanicity and rurality. For example, some population studies considered cities over 100,000 inhabitants their most urban category [37,38,53,54]. Others considered the examined country’s greatest city as their highest urbanicity value [33,39,55] and classified smaller settlements in different ways. The observed effect might be due to the specific settlement structure of each country but could limit the conclusions that can be drawn for countries with different settlement features. Besteher et al. [31] point out that settlements with a population over one million inhabitants might have a particular relevance especially when analyzing urban upbringing. Using the number of inhabitants as a score for urbanicity also assumes a continuous gradient for the effects of urban living [36]. The different ways of measurement result in difficulties in comparing the studies to each other regarding exposure [62]. It is not clear to what extent size or density of the urban habitat is a causal exposure or whether other variables mediate the effect. Wu et al. [40] tried with their Residential Environmental Assessment Tool to investigate more detailed how urban environments influence the health of residents. Likewise, the test administered cognitive performance tests show differences in focus and sensitivity. For example, the MMSE and MoCA are more oriented toward cognitive changes in aging, while tests such as the Wechsler Intelligence Test measure cognitive performance more universally. Consequently, comparisons here are also only useful to a limited extent. This emphasizes the urgency for universal definitions of urbanicity, as well as comparable measurement methods to clearly identify effects and exposures in science.

As discussed, different measurement periods of exposure were studied. While some experimental studies present scores to determine childhood urbanicity considering the participants first 15 life years [31,34,35,37,52] other studies used only current urbanicity as exposure. While this could be an exploratory advantage it also limits the comparability of some studies, since the experiences and exposures to urban spaces differ between age groups.

In addition, it was found that some of the identified studies used the same population samples. This might skew the results because the same participants might be considered in both studies. Both studies from Frissen et al. [34,35] used the exact same sample of Dutch adults. Hou et al. [44] and Xu et al. [47] studied the participants of the Chinese Longitudinal Healthy Longevity Survey. Xu, Vorderstrasse et al. [43] used data of the first Study on global Ageing and adult health (SAGE) Wave in China and India as well as Xu., Dupre et al. [41] that used the Chinese SAGE Wave and Xu et al. [42] which investigated the Indian SAGE Wave.

Public Health is a discipline that aims to keep the population healthy [63]. Consequently, it encompasses all organized efforts that prevent disease, promote health, and prolong life. The interdisciplinary field is characterized by a high degree of system involvement [64]. Thus, Public Health focuses on populations and their physical, economic, and political environments to identify salutogenetic and pathogenetic influences. In order to investigate the determinants of health in a holistic manner, the cooperation of different disciplines is required. Thus, within Public Health, disciplines such as sociology, political science, environmental science, or psychology and other disciplines contribute both methodologically and substantively. The understanding of health and disease within Public Health goes beyond the purely organic-biological attribution of causes and can be explained much more by bio-psycho-social causes [63]. In this context, Public Health claims to achieve an integrating effect between the medical-scientific and the social-behavioral-scientific paradigms [63]. The empirical representation of the interrelationships in real-world population-based studies is an essential component of Public Health research [21]. In contrast, neuroscience, which is also composed of various disciplines, has the primary goal of exploring the functioning of the nervous system, with a particular focus on the brain. The focus of neuroscientific knowledge is the experiment [65].

Of great scientific relevance for Public Health interventions is the identification of causal relationships [22]. Regarding the relationship between living in the city as an exposure and mental health as the outcome, there is a lack of scientific evidence that can precisely describe to what extent the measured effect can be attributed to a causal relationship or a selective relationship [23]. In order to approach the question, different study designs are needed that unambiguously describe the connection between outcome and exposure, whilst also remain transferable on a population level. Epidemiological studies, on which Public Health research is mostly based on, have already been able to sufficiently demonstrate a correlation between urban exposure and some mental illnesses that can be generalized to the population level [15,16]. However, since mostly observational studies are conducted, not all variables can be captured in this case and thus not all confounding variables can be controlled for. As a result, it is not possible to clearly determine the extent to which outcome and exposure are related. In contrast, the experimental studies can demonstrate the correlations relatively clear due to the artificial conditions in the experimental setting. However, generalizability of the results at the population level cannot simply be assumed (high internal validity, low external validity) [66]. Consequently, the typical study designs from Public Health and Neuroscience could complement each other at this point. The combination of both results could contribute to the identification of the underlying effects between urban environments and mental health.

In particular, longitudinal studies should be conducted to clarify the temporal relationship between exposure and outcome. Most of the studies included in this systematic review implemented a cross-sectional design, which has limited ability to prove causal associations between outcome and exposure due to being a one-time measurement [67]. In addition, all of the conducted longitudinal studies were retrospective studies, thus limiting the ability of each study to offer evidence about the causality of the investigated outcome [68]. Well-designed prospective longitudinal studies could further highlight which stage of one’s upbringing is particularly sensitive regarding urban or rural environments. This could be an important indication for preventive measures. Krabbendam et al. [62] also emphasize the need for longitudinal research to explore the impacts on developmental effects.

In addition to the methods described, such as brain scans or cognitive tests, other methods could be used to investigate effects. Ecological Momentary Assessments are a possible methodological approach to generate further knowledge regarding the underlying effects the urban environment has on individuals [62]. This method is based on the real time measurement of a participant’s behavior and their experience of the environment in contrast to retrospective judgments [69]. Ecological Momentary Assessment could be used to investigate outcomes such as mental well-being or loneliness, but also current stress caused by environmental factors (physiological und subjective parameters) as well as information processing parameter, e.g., using eye-tracking [70,71]. Some studies utilize mobile electroencephalography (EEG) in order to assess certain brain activity patterns that are associated with different environments [72,73]. Studies with a representative population sample and EEG-technology have the potential to combine aspects of Public Health as well as neuroscience.

The idea of Public Health focuses especially on the idea of equality in the distribution around health opportunities [64]. Consequently, it is important to identify disadvantaged or vulnerable groups. The studies included in this review examined different periods of investigation and groups of participants. In particular, urban upbringing has an influence on neuronal systems and can thus provide the field of Public Health with possible information that can help to design future prevention measures according to need. Buttazzoni et al. [21] also emphasize that the development in childhood and adolescence could be of great importance for mental health and as such should be assessed more frequently.

In addition to the limitations mentioned there are further aspects that should be acknowledged. No assessment of study quality was conducted due to heterogenicity in the study designs, as well as a variety of studied outcomes, which is restricting feasibility to assess all the included studies with the same assessment tool. In general, a quality assessment is not performed in scoping reviews [74]. This review does not consider studies published before the January 2016, in order to gather the latest evidence. Additionally, only studies published in English and German were considered. Thus, there might be relevant studies which were not included in this review.

## 5. Conclusions

The review was able to provide a first systematical approach in understanding the complex interactions of urban life on neural systems through the research between 2016 and 2021 and thus also contributes to exploring health opportunities and risks. Various neuronal and cognitive changes associated with living and growing up in urban areas became evident. By highlighting the topic from the perspectives of Public Health and Neuroscience, it became clear that the different disciplines can complement each other in order to identify underlying pathways between cities and mental health. Methodological synergies between experimental and population-based studies as well as perspectives of the disciplines were described. This demonstrates that the various strengths of the different approaches in combination could enhance Urban Health. The indispensable necessity of interdisciplinary solutions becomes apparent to enable healthier and more equitable living spaces for people, according to the Public Health idea. Further research should take up at this point and identify specific exposures and mechanisms that could be responsible for the effects shown. Before considering how neuroscientific findings can be fully integrated into the population context, precautions should first be taken. More attention to a standardization of the operationalization of urbanicity is necessary to support the comparability. Furthermore, studies of a longitudinal nature should be initiated to explore longterm causal effects to identify practical measures to enhance population health. In summary, the review provides an overview over studies conducted in the growing field of neurourbanism and can support future research.

## Figures and Tables

**Table 1:** Inclusion and exclusion criteria.

Inclusion Criteria	Exclusion Criteria
Measurement of at least one neuronal indicator, e.g., cognition, connection of neuronal systems	Studies which analyzed different urban exposures, e.g., air pollution
Measuring urbanization or urban upbringing	Subjective measuring of outcomes, e.g., stress
Urbanization as primary exposure	Studies regarding the effects of urbanicity on children
Publication between 2016 and 2021	Effects of urbanization on animals
Language: English and German Study Design: Empirical Studies	Published before 2016

## Data Availability

Not applicable.

## Supplementary Materials

The following supporting information can be downloaded at: https://www.mdpi.com/article/10.3390/ijerph20010688/s1, Table S1: Search String per Database; Table S2: Study characteristics.
