# Supplementary material for: Urbanicity—Perspectives from Neuroscience and Public Health: A Scoping Review
Source: Int J Environ Res Public Health. 2022 Dec 30;20(1):688. doi: 10.3390/ijerph20010688 (PMC9819040; doi:10.3390/ijerph20010688)
Supplement: Supplementary file 1 [file ijerph-20-00688-s001.zip › ijerph-2070236-supplementary Table S1.pdf]

Supplementary material

1 Table S1: Search String per Database

Database	Number of results	Search String
Embase	1.504 (12.11.2021)<br>1.566 (13.01.2022)	('urban dweller*':ab,ti OR 'city dweller*':ab,ti OR 'urban resident*':ab,ti OR 'urban population*':ab,ti OR 'urban population'/exp) AND ('urbanization'/exp OR 'population density'/exp OR 'urban*':ab,ti OR 'city living':ab,ti OR 'urban upbringing':ab,ti OR 'population density':ab,ti OR 'residence characteristics':ab,ti) AND ('schizophrenia':ab,ti OR 'depression':ab,ti OR 'depressive disorder*':ab,ti OR 'psychosis':ab,ti OR 'psychotic episode*':ab,ti OR 'psychotic disorder*':ab,ti OR 'anxiety disorder*':ab,ti OR 'brain':ab,ti OR 'stress':ab,ti OR 'cognitive function*':ab,ti OR 'cognition':ab,ti OR 'mental health':ab,ti OR 'schizophrenia'/exp OR 'depression'/exp OR 'psychosis'/exp OR 'anxiety disorders'/exp OR 'brain'/exp OR 'mental stress'/exp OR 'mental health'/exp) AND ([english]/lim OR [german]/lim) AND (2016:py OR 2017:py OR 2018:py OR 2019:py OR 2020:py OR 2021:py OR 2022:py)
MEDLINE (Pubmed)	1.421 (12.11.2021)<br>1.493 (13.01.2022)	(("urban dweller*"[Title/Abstract] OR "city dweller*"[Title/Abstract] OR "urban resident*"[Title/Abstract] OR "urban population"[MeSH Terms] OR "urban population*"[Title/Abstract]) AND ("urbanization"[MeSH Terms] OR "urban*"[Title/Abstract] OR "city living"[Title/Abstract] OR "urban upbringing"[Title/Abstract] OR "population density"[MeSH Terms] OR "population density"[Title/Abstract] OR "Residence Characteristics"[Title/Abstract] OR "Residence Characteristics"[MeSH Terms]) AND ("Schizophrenia"[MeSH Terms] OR "Schizophrenia"[Title/Abstract] OR "depressive disorder"[MeSH Terms] OR "Depression"[Title/Abstract] OR "depressive disorder*"[Title/Abstract] OR "psychotic disorders"[MeSH Terms] OR "Psychosis"[Title/Abstract] OR "psychotic episode*"[Title/Abstract] OR "psychotic disorder*"[Title/Abstract] OR "anxiety disorders"[MeSH Terms] OR "anxiety disorder*"[Title/Abstract] OR "brain"[MeSH Terms] OR "brain"[Title/Abstract] OR "stress, psychological"[MeSH Terms] OR "stress"[Title/Abstract] OR "cognitive function*"[Title/Abstract] OR "cognition"[Title/Abstract] OR "mental health"[MeSH Terms] OR "mental health"[Title/Abstract])) AND ((english[Filter] OR german[Filter]) AND (2016:2022[pdat]))
CINAHL	523 (12.11.2021)<br>552 (13.01.2022)	(TI "Urban dweller*" OR AB "Urban dweller*") OR (TI "City dweller*" OR AB "City dweller*") OR (TI "Urban resident*" OR AB "Urban resident*") OR (TI "Urban Population*" OR AB "Urban Population*") OR (MH "Urban Population") AND

		(TI "Urban*" OR AB "Urban*") OR (TI "City Living" OR AB "City Living") OR (TI "Urban upbringing" OR AB "Urban upbringing") OR (TI "Population density" OR AB "Population density") OR (TI "Residence Characteristics" OR AB "Residence Characteristics") OR (MH "Residence Characteristics") OR (MH "Population density") OR (MH "Urbanization") AND (TI "Schizophrenia" OR AB "Schizophrenia") OR (MH "Schizophrenia") OR (TI "Depression" OR AB "Depression") OR (TI "Depressive disorder*" OR AB "Depressive disorder*") OR (MH "Depression") OR (TI "Psychosis" OR AB "Psychosis") OR (TI "Psychotic episode*" OR AB "Psychotic episode*") OR (TI "Psychotic disorder*" OR AB "Psychotic disorder*") OR (MH "Psychotic disorders") OR (TI "Anxiety disorder*" OR AB "Anxiety disorder*") OR (MH "Anxiety disorders") OR (TI "Stress" OR AB "Stress") OR (MH "Stress, psychological") OR (TI "Cognitive function*" OR AB "Cognitive function*") OR (TI "cognition" OR AB "cognition") OR (TI "Mental health " OR AB "Mental health") OR (MH "Mental health") OR (TI "Brain" OR AB "Brain") OR (MH "Brain")<br>Limiters: Published Date: 20160101-20211231<br>Language: english
PsycInfo	318 (12.11.2021)<br>334 (13.01.2022)	(TI "Urban dweller*" OR AB "Urban dweller*") OR (TI "City dweller*" OR AB "City dweller*") OR (TI "Urban Population*" OR AB "Urban Population*") OR (MA "Urban Population*") AND (TI "Urban*" OR AB "Urban*") OR (TI "City Living" OR AB "City Living") OR (TI "Urban upbringing" OR AB "Urban upbringing") OR (TI "Population density" OR AB "Population density") OR (MA "Population density") OR (TI "Residence Characteristics" OR AB "Residence Characteristics") OR (MA "Residence Characteristics") OR (MA "Urbanization") AND (TI "Schizophrenia" OR AB "Schizophrenia") OR (MA "Schizophrenia") OR (TI "Depression" OR AB "Depression") OR (TI "Depressive disorder*" OR AB "Depressive disorder*") OR (MA "Depressive Disorder") OR (TI "Psychosis" OR AB "Psychosis") OR (TI "Psychotic episode*" OR AB "Psychotic episode*") OR (TI "Psychotic disorder*" OR AB "Psychotic disorder*") OR (MA "Psychotic disorders") OR (TI "Anxiety disorder*" OR AB "Anxiety disorder*") OR (MA "Anxiety disorders") OR (TI "brain" OR AB "brain") OR (MA "Brain")OR (TI "Stress" OR AB "Stress") OR (MA "Stress, psychological") OR (TI "Cognitive function*" OR AB "Cognitive function*") OR (TI "cognition" OR AB "cognition") OR (TI "Mental health " OR AB "Mental health ") OR (MA "Mental health")<br>Limiters - Publication Year: 2016-2021<br>Language: english, german

2
